# Active Traveling and Its Associations with Self-Rated Health, BMI and Physical Activity: A Comparative Study in the Adult Swedish Population

**DOI:** 10.3390/ijerph13050455

**Published:** 2016-04-28

**Authors:** Erik Berglund, Per Lytsy, Ragnar Westerling

**Affiliations:** Department of Public Health and Caring Sciences, Uppsala University, Box 564, Uppsala SE-75122, Sweden; per.lytsy@pubcare.uu.se (P.L.); ragnar.westerling@pubcare.uu.se (R.W.)

**Keywords:** active travel, commuting, self-rated health, BMI, physical activity, planning

## Abstract

Active traveling to a daily occupation means that an individual uses an active way of traveling between two destinations. Active travel to work or other daily occupations offers a convenient way to increase physical activity levels which is known to have positive effects on several health outcomes. Frequently used concepts in city planning and regional planning today are to create environments for active commuting and active living. Even then, little research has focused on traveling modes and subjective health outcomes such as self-rated health (SRH). This study aimed to explore and investigate associations between travel mode and health-related outcomes, such as self-rated health (SRH), body mass index (BMI) and overall physical activity, in an adult population in Sweden. A cross-sectional study was conducted in a randomly selected population-based sample (*n* = 1786, age 45–75 years); the respondents completed a questionnaire about their regular travel mode, demographics, lifestyle, BMI and SRH. Chi-square tests and logistic regressions found that inactive traveling was associated with poor SRH, a greater risk of obesity or being overweight and overall physical inactivity. In addition, lifestyle factors, such as choice of food and smoking habits, were associated with SRH, BMI and overall physical activity.

## 1. Introduction

In the Western world today, physical inactivity in everyday life is a critical issue [1]. According to the World Health Organization (WHO), the public health burden of physical inactivity is high and causes an estimated 600,000 deaths per year in Europe. Strong scientific evidence, presented since the 1950s [2,3], shows that physical activity promotes health, substantially lowers rates of many chronic diseases and decreases the risk of mortality. Physical activity prevents a number of disabling medical conditions such as coronary heart disease, hypertension, stroke, type 2 diabetes, metabolic syndrome, depression, cancer (some types) and all-cause mortality [4,5]. Physical inactivity has also significantly contributed to obesity reaching endemic proportions in both developed and developing countries [6]. The latest research also indicates that sitting time and sedentary lifestyle lead to worse health and all-cause mortality, independent of physical activity [7].

Traditionally, intervention strategies, such as promoting exercise at the individual level, have not been satisfactorily effective, and individual approaches to behavior changes are, in many cases, too small to make a difference at the population level [8]. Also, a growing body of evidence links the environment and the characteristics of neighborhoods to physical activity [9,10,11]. Therefore, there has been a shift in focus towards strategies that promote physical activity in everyday life such as making changes to the social and physical environment to improve conditions and remove barriers to active living [12,13].

Active traveling (AT), commuting or mobility to a daily occupation means that an individual uses an active (with energy expenditure) way of travel between two destinations: in practice, this usually means walking or bicycling. Theoretical analyses have been conducted in this field, and walking at 5 km/h over a smooth and level surface would create an energy expenditure of about 18 kJ/min [14]; bicycling in an urban area at 16 km/h would create an expenditure of about 36 kJ/min [15]. Active forms of traveling increase physical activity [16,17] and may reduce sitting time [18]. AT also is important for keeping a healthy body weight which is important for prevention [19,20,21]. AT has been directly associated with several health-related factors such as wellbeing [22], happiness [23], type 2 diabetes [24,25], cardiovascular risk [26], and all-cause mortality [17,27]. However, the association between traveling mode and all-cause mortality has not been strong in all studies [28]. Potentially, there may also exist individual adverse health effects such as a higher exposure to air pollution and a higher risk of traffic accidents when traveling in an active way; however, the health benefits of AT are estimated to be substantially larger than the risks relative to driving cars for individuals changing their transport mode [29,30]. Also, a systematic review concluded that effects of increased AT contributed to health benefits, which strongly outweighed the detrimental effects of traffic incidents and air pollution exposure, and AT provided substantial net health benefits, irrespective of geographical context [31].

Self-rated health (SRH) is one of the most widely used measures of personally perceived health. SRH, when measured via a one item question, is a robust predictor of several outcomes such as functional ability [32,33], returning to work [34], health care utilization [35], diseases [36], and mortality [37,38,39,40,41]. It has been shown that SRH is a more reliable predictor of future health than other, more objective measures [42]. SRH is a complex predictor; possible explanations for the functioning of SRH include its representation of an individual’s general perception of health, including biological, psychological and social dimensions. Several factors have been shown to be associated with SRH among them are: lifestyle habits such as smoking habits [43], and food intake [44].

Studies concerning commuting behavior have been based on a research approach that assumes that people who actively travel to work or other daily occupations acquire more physical activity and spend less time sitting, which leads to a lower risk of poor health outcomes [19,45]. Today, little research of this type has focused on travel mode and subjective health outcomes; a not fully explored and interesting consideration is how active travel relates to SRH in the adult population. This study aimed to explore and investigate associations between travel mode and health-related outcomes, such as SRH, BMI and overall physical activity, in a general adult population (aged ≥45 years) in Sweden. This study also aimed to discuss appropriate ways to manage travel mode and health-related behavior.

## 2. Materials and Methods

### 2.1. Sample

This is a cross-sectional, postal questionnaire study in an adult population-based sample (age 45–75). We randomly selected 3000 persons from the Swedish national population registry. Besides age, there were no inclusion criteria. The questionnaire was returned by post by 1786 individuals; fifty-one persons were not possible to reach or declined to participate and 1163 did not answer, making the response rate of the distributed questionnaires 60.6% (1786/2949). The study population consisted of slightly more women (54%) than men (46%). Data was collected from November 2013 to February 2014.

### 2.2. Explanatory Measures

The questionnaire contained 55 questions. The main data types and explanatory measures included are as follows.

Demographic data was collected by questions that assessed the respondent’s gender, age, educational level (categorized as compulsory school, secondary school or university) and occupation (categorized as student, working, work incapacity, unemployed, pensioner and sick leave).

Data on regular travel mode was assessed with the following question: “How do you usually travel when you go to work or to other activities outside your home?” Answers were collected on the following alternatives: 1. Most often I go by car; 2. Most often I go by bus or train; 3. Most often I go with a taxi service; 4. Most often I go by bicycle; and 5. Most often I walk. Travel mode was dichotomized into active travel (walking and bicycling) and inactive travel (alternatives 1 to 3).

Lifestyle habits were assessed with questions about smoking (never, current, previous) and choosing food for health reasons (very important, important to some degree, not important).

### 2.3. Health Outcome Measures

SRH was assessed with the question: “How do you rate your general state of health?”, and answers according to a five-point scale (very good, good, neither good nor poor, poor, very poor), and then dichotomized into either good (very good or good) or less than good (neither good nor poor, poor, very poor). In population studies, SRH is generally accepted by researchers as a valid measure for determining health status, and it is common to dichotomize SRH to demonstrate persons with not-good SRH [46].

Weight status variables were calculated from self-reported height and weight. BMI is a continuous variable defined as weight in kilograms divided by height in meters squared (kg/m^2^). BMI was dichotomized (cut-off score: 25) into normal weight (=0) and overweight or obese (=1) for the binary logistic regression analysis.

Overall physical activity was assessed as minutes of moderate physical activity per week, with the question: “How much time do you spend in a normal week doing moderately strenuous activities that make you warm?” Physical activity was then dichotomized into; reaching a sufficient amount of physical activity (150 min) per week (=0), or not (=1), according to WHO recommendations [47].

### 2.4. Statistical Analysis

The data was analyzed using Chi-square tests to compare differences in percentage distributions. Binary logistic regression models were used to analyze associations between travel mode, gender, age, education level, occupation, lifestyle factors and weight and the risk of poor SRH, less than sufficient physical activity, or being overweight. Multivariate analysis was performed to assess the sole association between traveling behavior and health outcomes. Variables were inserted stepwise in the multivariate regression analyses. Since the outcome measures used in this study have known associations to smoking and food intake, the multiple regression analyses were adjusted for smoking habits and choice of food in the full model. A Statistical Package for the Social Sciences (SPSS)^®^ version 22 (IBM SPSS Statistics for Windows, IBM Corp., New York, NY, USA) was used for all statistical analyses.

### 2.5. Ethical Consideration

The study (Project identification code DNR 2013/269) was approved by the regional Ethical Committee of Clinical Investigation in Uppsala (30 August 2013).

## 3. Results

The average age of the study population was 62 years, and secondary or equivalent school was the most common education level completed. The distribution of demographics and key variables in the study population are shown in Table 1.

### 3.1. Active and Inactive Traveling

There were 431 active travelers (25.9%) in the study population. More women (62.9%) than men (37.1%) were in the active travelers group (*p* ≤ 0.01). See Table 1.

### 3.2. SRH, BMI and Lifestyle, among Active and Inactive Travelers

A majority of the active travelers (78.8%) and inactive travelers (73.1%) reported good or very good global SRH. Less than good SRH was reported by 21.1% of the active travelers and 26.8% of the inactive travelers. The difference between active and inactive travelers’ SRH was significant (*p* ≤ 0.05).

On average, the BMI among active travelers was 25.2, and among the inactive travelers, the average BMI was 26.2. The majority (53.5%) of active travelers reported a BMI less that what is considered overweight and obese, and among the inactive travelers, 41.7% reported a BMI less that what is considered overweight and obese. The difference was significant (*p* ≤ 0.01). The two different traveling groups were explored with a particular focus on sex and weight. More males than females were overweight or obese, but the pattern in travel mode was the same. Inactive travelers also were overrepresented in the overweight or obesity group, regardless of whether they were men or women, and active travelers were overrepresented in the normal weight group.

In the total study population, 49.4% reached the recommended minimum of 150 min of physical activity a week. Among the active travelers 66.2% reached the recommended physical activity level, among inactive travelers, 45.5% reached the recommended physical activity level. The difference was significant (*p* ≤ 0.01).

Active and inactive travelers differed significantly regarding the choice of food for health reasons (*p* ≤ 0.05), but there were no differences in smoking habits between the two groups.

### 3.3. Logistic Regressions

SRH, travel mode, gender, age, education level, occupation and lifestyle factors were tested using binary logistic regression (Table 2). The significant differences between active and inactive travelers in SRH showed in the Chi-square test also remained consistent in the adjusted logistic regression models (odds ratio (OR) 1.42, 95% confidence interval (CI) 1.06–1.90).

The risk of being overweight or obese was tested via logistic regression (Table 3). Inactive traveling had a significant association with a higher risk of being overweight in the models adjusted for demographic and lifestyle factors (OR 1.43, 95% CI 1.13–1.81). Not choosing food for health reasons had the strongest association with being overweight or obese (OR 2.27, 95% CI 1.59–3.24).

Inactive traveling significantly increased the risk of being less than sufficiently physically active (defined by meeting physical activity recommendations of 150 min per week). In addition, in the models adjusted for demographic and lifestyle factors, inactive traveling increased the risk of insufficient overall physical activity (OR 1.77, 95% CI 1.40–2.23) (see Table 4).

## 4. Discussion

This study investigated associations of travelling mode and self-rated health, weight and reaching sufficiently physical activity in a general adult population aged 45 years or older. The main findings in this study were that poor SRH was more likely to be reported among inactive travelers than among active travelers. Having an inactive traveling behavior was associated with a higher risk of being overweight or obese. These results are consistent with other studies that have shown that inactive travelling is associated with lower SRH [48], however, previous studies have not adjusted for lifestyle factors such as smoking and choice of food. Demographic and lifestyle factors were associated with SRH, weight, and overall physical activity, and while the effect of travelling mode diminished when adjusting for these factors, it still remained statistically significant in the adjusted analysis. Previous studies have also found that active commuting provides a source for physical activity on a regular basis, and comparable results have been presented in several studies [9,49,50]. In a study population from Denmark, 50.6% met recommended levels of physical activity from active commuting alone [51]. The analyses concerning overweight and obesity show similarities in the results as previous studies [52].

The prevalence of AT was significantly higher among women than men, which has been seen in other population-based studies from Nordic countries [24,51]. There are large differences among countries in travel modes between men and women, where Nordic countries seem to be more gender-neutral than the US, Canada, and the UK [53]. There was no age difference between active and inactive travelers in this study; however, other studies have found that age may be a predictor of active commuting [54], and that younger individuals were more likely to use active ways of commuting [55]. These results may not have occurred in this study because it focuses only on adults over 45 years of age.

In this study population, the differences in SRH between active and inactive travelers were explicit but not very large. These results may have occurred because of a generally good SRH in the sample, however, from the perspective of public health, the differences due to traveling mode are certainly of substantial importance. The variables tested in the logistic model could explain 15% of the variance in SRH, which leaves 85% of unexplained variance in the outcome variable. This indicates that there are other factors not assessed in this model that affect SRH. There are several other factors known to have associations with SRH, and it may not be unexpected that SRH is one of the most inclusive health-reflecting measures available. SRH is a complex predictor, and early attempts to use SRH as only a proxy for disease burden have been unsuccessful [42]; even then, SRH seems to be affected by objective health to a great extent [36]. However, SRH is not considered to be easily affected by temporary situations [56].

The results of this study suggest that an approach managing traveling mode might impact SRH, regular overall physical activity, and the risk of being overweight. Several modifiable factors are known to be associated with activity–travel behavior, such as safety [57], attitudes, [58], attitudes of parents [59] travel-related infrastructure/connectivity [60], built environment [61,62], and mixed-use development that allow for shorter (more bikeable) trips [63]. Earlier studies have showed that traveling mode can preferably be affected through land-use and urban planning [64,65]. Many automobile trips today are short enough to be substituted by walking or biking, and there may exist a latent demand for more transportation-related activities [66]. These circumstances create opportunities to change transport behavior if the environment becomes suitable for active transport. However, the likelihood of positive health outcomes from AT will depend on the context within which individuals are travelling and how they travel [9]. For example, the length of the journey, intensity, frequency of active travel, route environment, nature of the terrain, risk perception, and levels of air pollution may influence health outcomes [67].

This study provides preliminary evidence that SRH should also be considered a potential positive outcome of AT among adults. Further investigations are needed regarding the associations made in this study to determine potential causality between travel mode and health. More research is also needed to understand fully how land-use planning efforts may contribute to better SRH.

### Strengths and Limitations

The strengths of this study include the community setting and the randomly selected approach. The response rate is reasonable according to what is expected in questionnaire studies.

AT is not defined consistently across studies, and the definition is dependent on what is considered normal in a certain setting [67]. In this study, traveling mode was based on the respondent’s most common way of travel to work or other activities outside the home. This approach was chosen to obtain a representative view of habits in everyday life. The downside to this measurement approach is that it is impossible to calculate how much energy travelers expend using different traveling modes. Another limitation is that, when asking participants about their main commuting mode, mixed-mode travelling modes were not captured, nor was travelling that relates to other domains of active transport (e.g., on leisure time).

This study also has some limitations worth noting in its design. Being a cross-sectional design, it is not possible to determinate the cause–effect relationship between travel mode and health outcomes. It can be discussed whether the directions of causality in the underlying assumption are appropriate, and reverse causality cannot be ruled out in this design. For example, people with poorer health and higher BMI may select inactive travelling modes, and healthier people may choose active forms of commuting. Further investigations need to focus on different sources of physical activity in more detail to explore if active travelers engage in more physical activity than non-active travelers. Longitudinal studies are needed to disentangle the relationship between different health outcomes and active and inactive travelling. More research is also needed to evaluate the role of potential confounding factors that were not accounted for in this study, as well as different types of active travelling and their relationship with SRH. One further study limitation is that reporting bias may exist due to the nature of self-reported data.

## 5. Conclusions

In this study, associations were found between traveling mode and SRH, BMI and overall physical activity. This study suggests that inactive adult travelers have an increased risk of poor SRH, being overweight and not being sufficiently physically active for 150 min per week, compared to commuters that use a physically active way of traveling. These findings add to existing evidence that increasing active commuting should be prioritized within transportation policies and urban planning efforts as they may prevent poor SRH and obesity, and increase regular physical activity.

## Figures and Tables

**Table 1:** Distribution of characteristics of active and non-active travelers.

Variables/Subgroups		Active Traveler (*n* = 431)	Inactive Traveler (*n* = 1231)	Total
Sex	Male	37.1 **	49.3 **	46.1
	Female	62.9 **	50.7 **	53.9
Age	Md, Mean (SD)	63, 62.3 (8.5)	62, 61.6 (8.5)	63, 61.8 (8.5)
Education	Compulsory school	28.7	27.8	28.0
	Secondary school or equal	33.6	38.4	37.1
	University	37.8	33.9	34.9
Occupation	Work full- or part-time	47.2	54.2	52.4
	Jobless or studding	4.0	2.8	3.1
	Pensioner	40.5	34.9	36.3
	Sick leave	8.4	8.1	8.2
Smoking habits	Never	49.2	46.6	47.3
	Previous	35.8	40.6	39.3
	Current	15.1	12.8	13.4
Choosing food for health reasons	Not important	9.4 *	12.3 *	11.5
	Important to some degree	24.4 *	29.5 *	28.2
	Very important	66.2 *	58.2 *	60.3
Total weekly physical activity	≤30 min	6.1 **	9.5 **	8.6
	30–60 min	8.9 **	13.7 **	12.4
	60–90 min	8.9 **	12.8 **	11.8
	90–150 min	15.5 **	18.6 **	17.8
	≥150 min	60.7 **	45.5 **	49.4
BMI	Normal	53.5 **	41.7 **	44.7
	Overweight	35.3 **	44.4 **	42.0
	Obesity	11.2 **	14.0 **	13.3
Self-rated health	Poor or very poor	3.9 *	6.5 *	5.9
	Neither good nor poor	17.2 *	20.3 *	19.5
	Good or very good	78.8 *	73.1 *	74.6

Figures as percentages if not stated otherwise. Pearson Chi-Square tests were used for distributions. Md = Median, SD = Standard deviation. * *p* ≤ 0.05; ** *p* ≤ 0.01.

**Table 2:** Results of logistic regression models of factors explaining the risk of poor SRH.

Variables		Crude OR 95% CI	Model 1 OR 95% CI	Model 2 OR 95% CI
Personal transportation	Traveling mode:			
	Active	1	1	1
	Inactive	1.37 * (1.04–1.79)	1.46 * (1.09–1.95)	1.42 * (1.06–1.91)
Demographic	Gender:			
	Male	1	1	1
	Female	0.90 (0.73–1.13)	0.86 (0.68–1.10)	0.95 (0.74–1.23)
	Age	1.02 ** (1.01–1.04)	1.00 (0.97–1.02)	0.99 (0.97–1.02)
	Education level:			
	University	1	1	1
	Secondary school or equal	1.71 ** (1.30–2.25)	1.51 ** (1.13–2.03)	1.42 * (1.05–1.92)
	Compulsory school	1.87 ** (1.40–2.49)	1.38 (1.00–1.90)	1.22 (0.89–1.69)
	Occupation:			
	Work full- or part-time	1	1	1
	Jobless or studding	2.35 ** (1.28–4.31)	2.08 * (1.10–3.93)	1.98 * (1.03–3.80)
	Pensioner	1.94 ** (1.52–2.49)	2.02 ** (1.32–3.09)	2.10 ** (1.36–3.24)
	Sick leave	8.93 ** (5.97–13.36)	9.12 ** (5.96–13.96)	8.75 ** (5.65–13.56)
Lifestyle	Smoking habits:			
	Never	1		1
	Previous	1.68 ** (1.31–2.14)		1.45 ** (0.11–1.90)
	Current	2.29 ** (1.64–3.18)		1.76 ** (1.22–2.54)
	Choosing food for health reasons:			
	Very important	1		1
	Important to some degree	1.80 ** (1.41–2.30)		1.88 ** (1.43–2.488)
	Not important	1.70 ** (1.21–2.40)		1.53 * (1.04–2.23)
Nagelkerke *r*^2^		-	0.13	0.15

SRH was dichotomized to lower than good (=1) and good or very good (=0); Odds ratio (OR), significance level and confidence interval (CI) for the binary logistic regressions. * *p* ≤ 0.05; ** *p* ≤ 0.01. Model 1 = Traveling mode + gender + age + education level + occupation; Model 2 = Model 1 + smoking habits + choosing food for health reasons.

**Table 3:** Results of logistic regression models of factors explaining the risk of obesity or being overweight.

Variables		Crude OR 95% CI	Model 1 OR 95% CI	Model 2 OR 95% CI
Personal transportation	Traveling mode:			
	Active	1	1	1
	Inactive	1.61 ** (1.29–2.01)	1.49 ** (1.18–1.87)	1.42 ** (1.13–1.80)
Demographic	Gender:			
	Male	1	1	1
	Female	0.50 ** (0.41–0.61)	0.51 ** (0.42–0.63)	0.57 ** (0.46–0.71)
	Age	1.01 (1.00–1.02)	1.00 (0.99–1.02)	1.00 (0.98–1.02)
	Education level:			
	University	1	1	1
	Secondary school or equal	1.65 ** (1.32–2.06)	1.52 ** (1.19–1.93)	1.51 ** (1.19–1.93)
	Compulsory school	2.01 ** (1.58–2.57)	2.05 ** (1.57–2.69)	2.03 ** (1.54–2.68)
	Occupation:			
	Work full- or part-time	1	1	1
	Jobless or studding	0.78 (0.45–1.36)	0.78 (0.44–1.41)	0.72 (0.39–1.31)
	Pensioner	1.06 (0.86–1.30)	0.85 (0.60–1.20)	0.84 (0.59–1.20)
	Sick leave	1.32 (0.92–1.91)	1.21 (0.81–1.79)	1.19 (0.79–1.78)
Lifestyle	Smoking habits:			
	Never	1		1
	Previous	1.25 * (1.02–1.54)		1.18 (0.94–1.48)
	Current	0.81 (0.60–1.08)		0.64 ** (0.46–0.88)
	Choosing food for health reasons:			
	Very important	1		1
	Important to some degree	1.80 ** (1.45–2.25)		1.63 ** (1.28–2.07)
	Not important	2.34 ** (1.69–3.22)		2.27 ** (1.59–3.24)
Nagelkerke *r*^2^		-	0.08	0.11

Risk of obesity or being overweight was dichotomized to having a BMI over 24.9 (=1) and under 25 (=0); Odds ratio (OR), significance level and confidence interval (CI) for the binary logistic regressions; * *p* ≤ 0.05, ** *p* ≤ 0.01; Model 1 = Traveling mode + gender + age + education level + occupation; Model 2 = Model 1 + smoking habits + choosing food for health reasons.

**Table 4:** Results of logistic regression models of factors explaining risk of less than 150 min a week of physical activity.

Variables		Crude OR 95% CI	Model 1 OR 95% CI	Model 2 OR 95% CI
Personal transportation	Traveling mode:			
	Active	1	1	1
	Inactive	1.85 ** (1.48–2.32)	1.80 ** (1.43–2.27)	1.77 ** (1.40–2.23)
Demographic	Gender:			
	Male	1	1	1
	Female	0.86 (0.72–1.06)	0.96 (0.79–1.18)	1.10 (0.89–1.36)
	Age	1.00 (0.98–1.01)	1.00 (0.98–1.02)	1.00 (0.98–1.02)
	Education level:			
	University	1	1	1
	Secondary school or equal	1.38 ** (1.11–1.73)	1.43 ** (1.13–2.11)	1.32 * (1.04–1.68)
	Compulsory school	1.52 ** (1.20–1.93)	1.61 * (1.24–2.10)	1.44 ** (1.10–1.89)
	Occupation:			
	Work full- or part-time	1	1	1
	Jobless or studding	1.16 (0.67–2.01)	1.03 (0.58–1.83)	0.92 (0.51–1.65)
	Pensioner	0.85 (0.70–1.05)	0.80 (0.57–1.13)	0.79 (0.56–1.12)
	Sick leave	1.06 ** (0.74–1.51)	0.97 (0.67–1.42)	0.93 (0.63–1.38)
Lifestyle	Smoking habits:			
	Never	1		1
	Previous	1.12 (0.91–1.37)		1.13 (0.91–1.41)
	Current	1.59 ** (1.18–2.14)		1.37 (0.99–1.89)
	Choosing food for health reasons:			
	Very important	1		1
	Important to some degree	2.16 ** (1.74–2.69)		1.98 ** (1.56–2.50)
	Not important	2.43 ** (1.78–3.32)		2.39 ** (1.70–3.34)
Nagelkerke *r*^2^		-	0.04	0.08

Risk of less than 150 min a week of physical activity was dichotomized to less than sufficiently physical activity (=1) and reaching sufficiently physical activity or more (=0); Odds ratio (OR), significance level and confidence interval (CI) for the binary logistic regressions; * *p* ≤ 0.05, ** *p* ≤ 0.01; Model 1 = Traveling mode +gender +age +education level +occupation; Model 2 = Model 1 +smoking habits + choosing food for health reasons.
